# Dopaminergic Neurons in the Zebrafish Subpallium Belong to the Extended Medial Amygdala

**DOI:** 10.1002/cne.70079

**Published:** 2025-08-05

**Authors:** Daniel Armbruster, Thomas Mueller, Wolfgang Driever

**Affiliations:** ^1^ Institute for Biology I, Faculty of Biology University of Freiburg Freiburg Germany; ^2^ MeInBio Research Training Group University of Freiburg Freiburg Germany; ^3^ CIBSS – Centre for Integrative Biological Signalling Studies University of Freiburg Freiburg Germany; ^4^ Department of Biology Montclair State University Montclair New Jersey USA

## Abstract

The amygdala is a heterogeneous, multinuclear telencephalic structure critical for motivated and emotion‐related behaviors in vertebrates. In ray‐finned fish (Actinopterygii) like the teleost zebrafish, a telencephalic outward‐growing process called eversion makes defining amygdaloid territories particularly challenging. Teleosts are also peculiar in that they develop numerous dopaminergic (DA) neurons in the subpallium, while in tetrapods, such populations are less prominent or appear only transiently. To shed light on the organization of the amygdala in teleosts, we pursued an evolutionary developmental approach focusing on the topological origin of subpallial DA neurons. Specifically, we analyzed the distribution of tyrosine hydroxylase (Th) in conjunction with expression patterns of *pax6a+b*, *isl1a*, *nkx2.1*, *lhx8a*, *otpa+b*, and *calb2a* as markers of different telencephalic subdivisions in brains of 5‐ and 30‐day‐old zebrafish (*Danio rerio*, Teleostei). Our data show that the previously identified dorsalmost division of the ventral telencephalon (Vdd) needs to be subdivided into an anteroventral *pax6a/b*‐positive portion (Vdd1) and a posterodorsal *pax6a/b*‐negative portion (Vdd2). This *pax6a*‐negative Vdd2 portion develops into the extended medial amygdala (EMeA), including the DA population adjacent to the pallial–subpallial border. Our results also show that the EMeA DA neurons form a heterogeneous group of amygdaloid neurons because they differentially express *calb2a* and *sst7*. Our work sheds light on the early evolution and development of the amygdala and provides a foundation for functional analysis of the newly defined DA subtypes of the extended amygdala in zebrafish.

## Graphical abstract

We identify directly adjacent to the pallial–subpallial boundary a *pax6a*‐negative dorsal Vdd2 portion of the zebrafish larval subpallium that develops into the extended medial amygdala (EMeA). Zebrafish subpallial dopaminergic neurons that locate to Vdd2 belong to the EMeA and form a heterogeneous group differentially expressing *calbindin2a* and *cortistatin/sst7*.

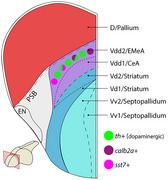

**Abbreviations**
(v/d)LGE: (ventral/dorsal) lateral ganglionic eminence
BST: bed nucleus of the stria terminalis
BSTa: anterior part of BST (in zebrafish)
CeA: central amygdala
EA: extended amygdala
EMeA: extended medial amygdala
EN: entopeduncular nucleus
MeA: medial amygdala
MeAp: posterior medial amygdala
MGE: medial ganglionic eminence
OB: olfactory bulb
ORR: optic recess region
PSB: pallial–subpallial border
SPV: supraoptoparaventricular area
Vc: central division of the ventral telencephalon
Vd: dorsal division of the ventral telencephalon
Vdd: dorsalmost division of the ventral telencephalon
Vp: posterior division of the ventral telencephalon
Vs: supracommissural division of the ventral telencephalon
Vv: ventral division of the ventral telencephalon

## 1 Introduction

Dopaminergic (DA) neurons form several highly conserved neuromodulatory systems in the vertebrate brain, including those of the retina, olfactory bulb (OB), hypothalamus, and ventral diencephalon (Smeets and Gonzalez 2000; Yamamoto and Vernier 2011). In contrast, there are severe differences between tetrapods and teleosts with regard to mesodiencephalic and subpallial DA groups, with the latter being the focus of the current study in the zebrafish (*Danio rerio*) model.

Amphibians, birds, and mammals show prominent mesodiencephalic DA systems, two of which project to cortical and limbic structures, and are involved in the regulation of learning and memory processes, reward system, and fear responses, while the third projects to the dorsal striatum, contributing to the modulation of movement control pathways of the basal ganglia (Bjorklund and Dunnett 2007; Iversen and Iversen 2007; Bromberg‐Martin et al. 2010). Strikingly, these substantial populations of mesodiencephalic DA neurons are not conserved in all vertebrate taxa, since they are not present in lamprey and ray‐finned fish (Actinopterygii) (Pierre et al. 1997). For the widely used teleost zebrafish model, the absence of prominent midbrain DA neuronal groups has been documented (Holzschuh et al. 2001; Kaslin and Panula 2001; Filippi et al. 2010; Yamamoto et al. 2010). However, recently, sparse cells that weakly express tyrosine hydroxylase (Th), a marker for catecholaminergic neurons, have been detected in the midbrain of various teleosts (Lopez et al. 2019; Lozano et al. 2019; Borgonovo et al. 2021; Altbürger et al. 2023). In zebrafish, the midbrain Th‐positive cells do not express other markers required for DA signaling (Altbürger et al. 2023), indicating that they may be nonfunctional with regard to DA neuromodulation. Several species of cartilaginous fish (Chondrichthyes) possess DA neurons in areas corresponding to the ventral tegmental area and substantia nigra (Stuesse et al. 1994; Meredith et al. 1999), suggesting that teleosts may have secondarily lost midbrain DA neurons.

In contrast to tetrapods, teleosts evolved a well‐developed group of DA neurons in the subpallium (Guo et al. 1999; Holzschuh et al. 2001; Rink and Wullimann 2002; Filippi et al. 2010; Yamamoto and Vernier 2011). Interestingly, also in the striatum of rodents and primates, including humans, TH‐immunoreactive neurons have been identified that were classified as GABAergic TH interneurons (THINs) (Dubach et al. 1987; Tashiro et al. 1989; Porritt et al. 2000; Ibanez‐Sandoval et al. 2010). However, further characterization of THINs in rodents revealed that they neither express other proteins required for functional DA neuromodulation nor release dopamine upon optogenetic activation (Xenias et al. 2015). The presence of striatal THINs and the mesodiencephalic systems in mammals, compared to the subpallial DA system and remnant midbrain *th*‐expressing neurons in zebrafish, suggests a divergent evolution of DA systems in vertebrates. This raises the question about potential sources of dopamine in the zebrafish pallium and subpallium. Apart from a few projections of a small subset of the diencephalic DA cells in the posterior tuberculum, no extratelencephalic DA input into the zebrafish telencephalon proper has been shown (Rink and Wullimann 2001; Tay et al. 2011). Due to their extensive local intratelencephalic arborizations (Tay et al. 2011), the subpallial DA neurons could form an alternative source for telencephalic DA modulation in zebrafish. Like THINs of mammals, the zebrafish subpallial DA neurons have a GABAergic co‐transmitter type, consistent with recent findings in goldfish (Filippi et al. 2014; Tibi et al. 2023). Unfortunately, so far, there is limited knowledge of anatomical localization, function, and connectivity of these subpallial DA cell populations. Due to their topographical localization in the dorsal and central subpallium (Vd, Vc), these cells in zebrafish have been viewed as part of the teleostean striatum (Tay et al. 2011). Recently, however, we proposed that the subpallial DA neurons of zebrafish are located in a region of the teleostean equivalent of the subpallial extended amygdala (EA) that we specified as the anterior bed nucleus of the stria terminalis (BSTa) (Porter and Mueller 2020).

The amygdala has mostly been investigated in tetrapods, revealing that it is composed of a number of pallial and subpallial nuclei with interspersed cell types of different developmental origins, including tangentially migrated prethalamic, preoptic, and hypothalamic ones (Moreno and Gonzalez 2007; Aerts and Seuntjens 2021; Medina et al. 2023). These publications show that in tetrapods, cell types within the different nuclei can substantially differ in their expression profile and connectivity, and that most of the subpallial EA, which consists of the medial amygdala (MeA), the central amygdala (CeA), and the bed nucleus of the stria terminalis (BST), originates from the lateral, medial, and caudal ganglionic eminences (LGE, MGE, and CGE). When discussing the neuroanatomy of the teleostean amygdala, one must consider that the concept of ‘the amygdala’ has shifted over time and evolved with an increased understanding of the prosomeric organization of vertebrate forebrains. In addition, the telencephalon of teleosts differs substantially from that of mammals due to its outward growing process (eversion) (Wullimann and Mueller 2004; Nieuwenhuys 2009; Mueller et al. 2011; Folgueira et al. 2012; Furlan et al. 2017). Based on topology and molecular marker expression, a prosomeric ground plan of the zebrafish amygdala has been identified that defines a number of homologs to mammalian amygdaloid nuclei (Porter and Mueller 2020). These include regions corresponding to CeA and MeA amygdaloid nuclei, as well as the teleostean equivalent of the BST in the dorsal (Vd(d)), central (Vc), posterior (Vp), and supracommissural (Vs) divisions of the ventral telencephalon.

One controversially discussed and often overlooked territory represents the *isl1*‐negative dorsalmost division of the ventral telencephalon (Vdd) described for carp‐like (cyprinid) goldfish (Northcutt 2006). Prior studies have considered the Vdd region in zebrafish a dorsal striatal region (Ganz et al. 2012). However, the dorsal striatum (caudate and putamen) of mammals is defined as a derivative of the *Isle*t‐1‐positive ventral LGE (vLGE), and the majority of dorsal striatal neurons develop from *Islet‐1*‐expressing precursor cells. We therefore postulated that the teleostean Vdd region represents an extended portion of the dorsal LGE (dLGE) that has not been identified or may differ from the one in tetrapods (Porter and Mueller 2020). Better knowledge of Vdd development is also critical for understanding the morphogenetic processes underlying telencephalic eversion and, ultimately, the developmental constraints of amygdala evolution in vertebrates.

To better characterize the subpallial DA system in zebrafish and the evolution of the amygdala, we analyzed the distribution of Th‐immunoreactive neurons in comparison to markers for distinct subpallial territories at larval stages. Our analyses of transcription factor expression locate these DA neurons to the dorsalmost compartment of the subpallium, directly adjacent to the pallial–subpallial border (PSB). The DA neurons are positioned in a domain of *calb2a* expression, characteristic of the extended medial amygdala (EMeA). Indeed, a DA subpopulation expresses *calb2a*, demonstrating that the zebrafish subpallial DA system belongs to the EMeA. Within the EMeA, a subpopulation of DA neurons expresses *sst7*, revealing a diversity of this DA group. We present a detailed anatomical model for the EMeA DA neurons in the 5‐day postfertilization (dpf) larval brain. Our findings provide a basis to investigate the development and function of the intratelencephalic DA system evolved in the subpallial amygdala of teleosts.

## 2 Material and Methods

### 2.1 Zebrafish Strains, Maintenance, and Specimen Preparation

Wildtype zebrafish of the ABTL strain (www.ZFIN.org [ID ZDB‐GENO‐031202‐1]) were kept under standard conditions (Westerfield 2000). The following mutant alleles were used in this study: *otp^m866^* (Ryu et al. 2007) (RRID:ZFIN_ZDB‐ALT‐070531‐1) and *otpb^sa135^* (Fernandes et al. 2013) (RRID:ZFIN_ZDB‐ALT‐100506‐15). Genotyping of larvae was performed on DNA extracted from tail‐clips of fixed and stained larvae according to the published PCR protocols (Ryu et al. 2007; Fernandes et al. 2013).

Embryos obtained by natural breeding were raised at 28.5°C in 3 g/L Red Sea salt (Red Sea Aquatics Ltd) and staged according to Kimmel et al. (1995). To inhibit pigmentation, 0.2 mM *n*‐phenylthiourea was added to the media. At 5 dpf, larvae were fixed in 4% paraformaldehyde (PFA) in PBST (PBS + 0.1% Tween 20) at 4°C overnight. After fixation, fish were washed in PBST and brains were dissected. Dissected brains were stored in 100% methanol at −20°C until needed. Thirty‐day postfertilization fish were treated with MS‐222 (0.300 mg/mL) until cardiac arrest, followed by decapitation. Subsequently, the brains were dissected, fixed, and stored as described for 5‐dpf larvae. All experiments were carried out in accordance with the German Animal Welfare Act.

### 2.2 Cloning of cDNA Fragments and In Situ Probe Synthesis

To extract RNA from 3‐dpf embryos of the ABTL strain, the Qiagen RNeasy Mini Kit (Qiagen, ID: 74004) was used according to the manufacturer's instructions. cDNA was synthesized using the SuperScript III Reverse Transcriptase Kit (Invitrogen, 18080093) using oligo(DT)_20_ primer according to the manufacturer's instructions. Coding sequence fragments of *calb2a* (ENSDART00000060160.5), *lhx8a* (ENSDART00000019078.5), *sst7* (ENSDART00000047378.7; recently renamed to *sst6.1*), and *tbr1b* (ENSDART00000006612.7) were amplified using MyTaq DNA Polymerase (Bioline, BIO‐21107) and the following primers:

*calb2a* fwd: 5ʹ‐GAAATACGATACAGACCGCAGC‐3ʹ
*calb2a* rev: 5ʹ‐GCCATGATGCTTTGCTTGTAAC‐3ʹ
*lhx8* fwd: 5ʹ‐AGCCTGTCATACTGGACAATG‐3ʹ
*lhx8* rev: 5ʹ‐CATGCTGTCCTCTGACCTGA ‐3ʹ
*sst7* fwd: 5ʹ‐ CAGCACAAAGTAGGGAGTTGAG‐3ʹ
*sst7* rev: 5ʹ‐ AGAGTAAGTCCACGGAGACC‐3ʹ
*tbr1b* fwd: 5ʹ‐ TGGCTACCCAAACGCACAAG‐3ʹ
*tbr1b* rev: 5ʹ‐ GCGGAGGAAAACTGGTAGAAAG‐3ʹ

Expected PCR product sizes were identified by agarose gel electrophoresis and cloned into the pCRII‐TOPO plasmid (Invitrogen, K461020), and their cDNA sequence was verified by sequencing. Probes used in this study are given in Table S1.

### 2.3 Fluorescent Whole Mount In‐Situ Hybridization (WISH)

WISH was performed as described previously with minor modifications (Filippi et al. 2007; Ronneberger et al. 2012). Brains were rehydrated in decreasing concentrations of methanol, washed with PBST, and incubated with 1% hydrogen peroxide in PBST for 30 min at room temperature (RT). After several PBST washing steps, permeabilization was performed by incubating in 4% Triton X‐100 (AppliChem, A1388) in PBST for 60 min at RT. After rinsing with PBST, brains were postfixed in 4% PFA/PBST for 30 min and washed several times with PBST. For prehybridization, brains were incubated in hybridization buffer (50% formamide, 5× SCC, 50 µg/mL heparin, 5 mg/mL torula RNA, 0.1% Tween 20) at 65°C for at least 4 h. DIG‐ and DNP‐labeled probes were diluted in hybridization buffer, and hybridization was performed overnight at 65°C. The following day, the unbound probe was washed out using decreasing concentrations of formamide in SSC at 65°C. After washing in TNT (100 mM Tris‐HCl pH 7.5, 150 mM NaCl, 0.5% Tween 20) at RT, brains were incubated with 1% Blocking Reagent (Roche, #1096176) for 2 h and incubated with 1:400 Anti‐Digoxigenin‐POD (Roche, #11207733910; RRID:AB_514500) overnight at 4°C. Brains were then washed several times with TNT and stained for 45 min with a tyramide‐Alexa 488 or 555 working solution according to the manufacturer's instructions (Invitrogen, B40953/B40955). After several TNT washes, we proceeded with ant‐iTh immunohistochemistry or continued with the staining of the DNP probe as follows. The peroxidase of the Anti‐Digoxigenin‐POD antibody was inactivated by incubation in 1% hydrogen peroxide in TNT for 30 min at RT. Brains were washed several times in TNT, followed by blocking as described above and incubation in 1:100 Anti‐DNP‐HRP conjugate (Akoya Biosciences, TS‐000400) overnight at 4°C. On the following day, brains were washed in TNT, followed by incubation in tyramide‐Alexa 488 or 555 working solution for 90 min, and washed several times in TNT. We proceeded with the anti‐Th immunohistochemistry as described below.

### 2.4 Immunohistochemistry

Brains previously stained by fluorescent in situ hybridization were equilibrated in PBTD (1% DMSO in PBST) and incubated in antibody blocking solution (5% goat serum [Sigma Aldrich, #G9023], 1% Blocking Reagent [Roche, #1096176], 1% BSA [Sigma Aldrich #A6003]) for 2 h at RT. Brains were incubated overnight at 4°C with 1:500 polyclonal rabbit‐anti‐Th primary antibody in blocking solution (Ryu et al. 2007). This antibody (RRID:AB_2631248) is directed against the peptide encoded by nucleotides 274–1097 of the zebrafish *th* gene (GenBank accession No. NM_131149). The antibody recognizes the protein encoded by *th* (ENSDARG00000030621), but not the protein encoded by *th2* (ENSDARG00000038384) (Filippi et al. 2010). On the following day, brains were washed several times with PBTD containing 200 mM KCl. After overnight incubation with 1:1000 secondary goat‐anti‐rabbit IgG Alexa 633 (Invitrogen, A21070; RRID:AB_2535731) in antibody blocking solution, brains were washed in PBTD containing 200 mM KCl, followed by several PBST washing steps, and stored in 80% Glycerol/PBST at 4°C until confocal imaging.

### 2.5 Specimen Clearing and Hybridization Chain Reaction (HCR)

Dehydrated 30‐dpf brains were rehydrated in decreasing concentrations of methanol in PBST and washed several times in PBST. Afterward, brains were incubated for 45 min in DEEP‐Clear Solution‐1.1 (Pende et al. 2020) at 37°C. After rinsing with PBST, brains were postfixed with 4% PFA/PBST for 20 min at RT. After several PBST washing steps, multiplexed HCR was performed according to the manufacturer's protocol v3.0 (Molecular Instruments). HCR probes for *calb2a* (ENSDART00000060160.5) and *th* (ENSDART00000040410.7) detection are given in Table S2.

### 2.6 Imaging and Figure Preparation

Samples for imaging were mounted in 80% Glycerol/20% PBST/1.5% low‐melting Agarose (Biozym Scientific, 850080). Imaging was performed on the Zeiss LSM 880 confocal microscope (Life Imaging Center, University of Freiburg) using the LD LCI Plan‐Apochromat 25×/0.8 NA Imm Corr DIC M27 objective and the Zeiss ZEN Black 2.3 software (RRID:SCR_018163). The 30‐dpf brain stack presented in Figure 6c was recorded in confocal mode at a resolution of 512×512 pixels, with a pixel size of 0.95 × 0.95 µm and a *z*‐plane thickness of 2 µm. The stack used for the sagittal reconstruction of Figure 6e was recorded in confocal mode at a resolution of 1024×1024 pixels, with a pixel size of 0.33 × 0.33 µm and a *z*‐plane thickness of 1.3 µm. The stacks used in Figure 6f,g were recorded in confocal mode at a resolution of 1024×1024 pixels, with a pixel size of 0.66 × 0.66 µm and a *z*‐plane thickness of 2 µm. The confocal mode detector range for Alexa 488 was set to 499–553 nm, for Alexa 555 to 570–624 nm, and for Alexa 633 to 642–686 nm. All other images were acquired using the Zeiss FastAiryScan Mode with a typical resolution between 1012×1012 and 1028×1028 pixels, a pixel size of 0.30 × 0.30 µm, and an optimal *z*‐plane thickness (calculated by the Zeiss imaging software) between 0.57 and 0.65 µm. The detector settings were set prior to each microscopy session. For the recording of Alexa 488, it ranged between 515 and 516 nm, for Alexa 555 between 590 and 595 nm, and for Alexa 633 between 645 and 654 nm. Afterward, acquired image stacks were processed using the AiryScan Processing Tool of the ZEN Black 2.3 software. For each analysis, two to three brains were documented, including one AiryScan Stack of higher resolution (exact numbers for each analysis are provided in the respective figures). If horizontal and transversal views of the same staining are shown, they were obtained from the same embryo, which was repositioned and reembedded before imaging. Thus, the positions of the corresponding planes depicted in the figures are approximate, based on the respective complementary views, which were computationally reconstructed for this purpose using the reslicing plugin (Identifier: legacy:ij.plugin.Slicer) of the Fiji distribution (RRID:SCR_002285) of ImageJ (Schindelin et al. 2012). The scales were generated from the metadata of the ZEN image stacks using Fiji and then adjusted in thickness for visibility using Adobe Illustrator CC. Unless otherwise mentioned in the figure legends, image brightness was linearly adjusted using Fiji. For better visualization, the brightness of the blowups was adjusted linearly as needed. The sagittal views presented in Figures 4c and 6e were generated from horizontal stacks with the computational reslicing plugin (Identifier: legacy:ij.plugin.Slicer) in Fiji. Supporting Information Videos were assembled using Adobe Premiere Pro 2025 (RRID:SCR_021315). Figures were assembled, and the schematic brain model was generated using Adobe Illustrator CC (RRID:SCR_010279).

## 3 Results

### 3.1 Subpallial DA Neurons Have a GABAergic Cotransmitter Type in 5‐dpf Zebrafish Larvae

**FIGURE 1:**
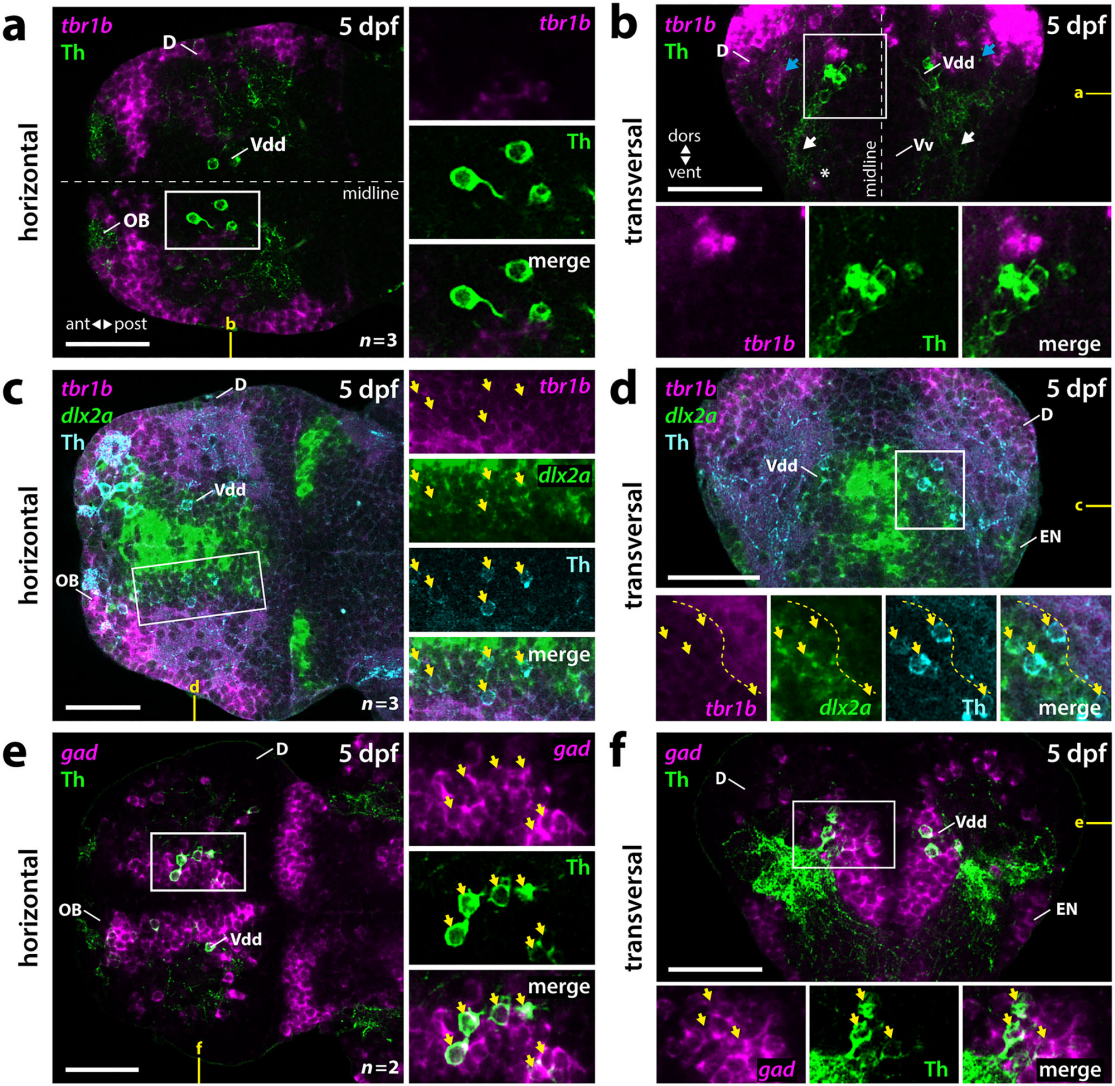
Validation of the subpallial localization of telencephalic DA neurons. (a–f) Fluorescent in situ hybridization combined with anti‐Th immunofluorescence in dissected 5‐dpf zebrafish brains. (a, b) The pallial (D) marker *tbr1b*. (a) Horizontal plane at the level of Vdd as marked in yellow in (b). (b) Transversal plane at a precommissural level as marked in yellow in (a). White asterisk marks scattered septal *tbr1b*‐positive cells in Vv. Blue arrows mark Th‐immunoreactive fibers in D. White arrows mark Th‐immunoreactive fibers in lateral parts of the ventral subpallium. (c, d) Staining for *tbr1b* and, as marker for subpallial regions, *dlx2a* shows that subpallial DA neurons are located at the PSB in Vdd. Yellow arrows mark cells that express *dlx2a* and are immunoreactive for anti‐Th. (c) Horizontal plane at the level of Vdd as marked in yellow in (d). (d) Transversal plane at precommissural levels marked in yellow in (c). Yellow dotted line marks the border to the *tbr1b* D. (e, f) Combined probes for *gad1a*, *gad1b*, and *gad2* together mark all GABAergic neurons, and in combination with immunostaining for Th confirm the GABAergic cotransmitter type of subpallial DA neurons. Yellow arrows mark cells positive for *gad* and immunoreactive for anti‐Th. (e) Horizontal plane at the level of Vdd at the position marked in yellow in (f). (f) Transversal plane at precommissural levels marked in yellow in (e). (a–f) White boxes mark the areas presented in the blowups. *n*, number of analyzed brains per experiment; ant, anterior; D, dorsal telencephalic area (pallium); dors, dorsal; dpf, days postfertilization; EN, entopeduncular nucleus; OB, olfactory bulb; post, posterior; Vdd, dorsalmost division of the ventral telencephalon; vent, ventral; Vv, ventral division of the ventral telencephalon. Scale bars = 50 µm.

In the 5‐dpf zebrafish telencephalon, Th‐immunoreactive DA neurons can be detected in Vd(d) and Vp, directly adjacent to *tbr1b‐*expressing regions likely corresponding to the medial (Dm) and posterior division (Dp) of the pallium (Figure 1a,b). As previously reported, the subpallial DA neurons project locally and into lateral parts of the ventral subpallium (Vv) (Figure 1b, white arrows) (Tay et al. 2011). Th‐immunoreactive innervation can also be detected in the *tbr1b‐*positive regions of D (Figure 1b, blue arrows). Scattered *tbr1b‐*expressing cells can be detected in Vv, which might represent septal cells of pallial origins as described previously (Figure 1b, white asterisk) (Mione et al. 2001; Mueller et al. 2008; Filippi et al. 2012; Ganz et al. 2012). The subpallial DA neurons are positioned medially adjacent to *tbr1b‐*positive pallial regions and thus are located in the most dorsolateral portion of the *dlx2a‐*positive Vdd (Figure 1c,d). Dlx2 marks the ventral forebrain, including subpallial areas, throughout vertebrates (Puelles et al. 1999; Mueller et al. 2008; Moreno et al. 2009).

We observed coexpression of Th and *gad* (combinatorial staining of *gad1a*, *gad1b*, and *gad2*) in the subpallium at 5 dpf (Figure 1e,f), confirming the GABAergic cotransmitter type of these neurons (Filippi et al. 2014). The laterally displaced *dlx2a‐* and *gad2*‐positive areas likely correspond to the entopeduncular nucleus (EN) (Figure 1d,f) (Solek et al. 2017; Wullimann 2022).

### 3.2 Subpallial DA Neurons Are Located Dorsolateral of *nkx2.1*‐ and *lhx8a*‐Positive MGE Derivatives

**FIGURE 2:**
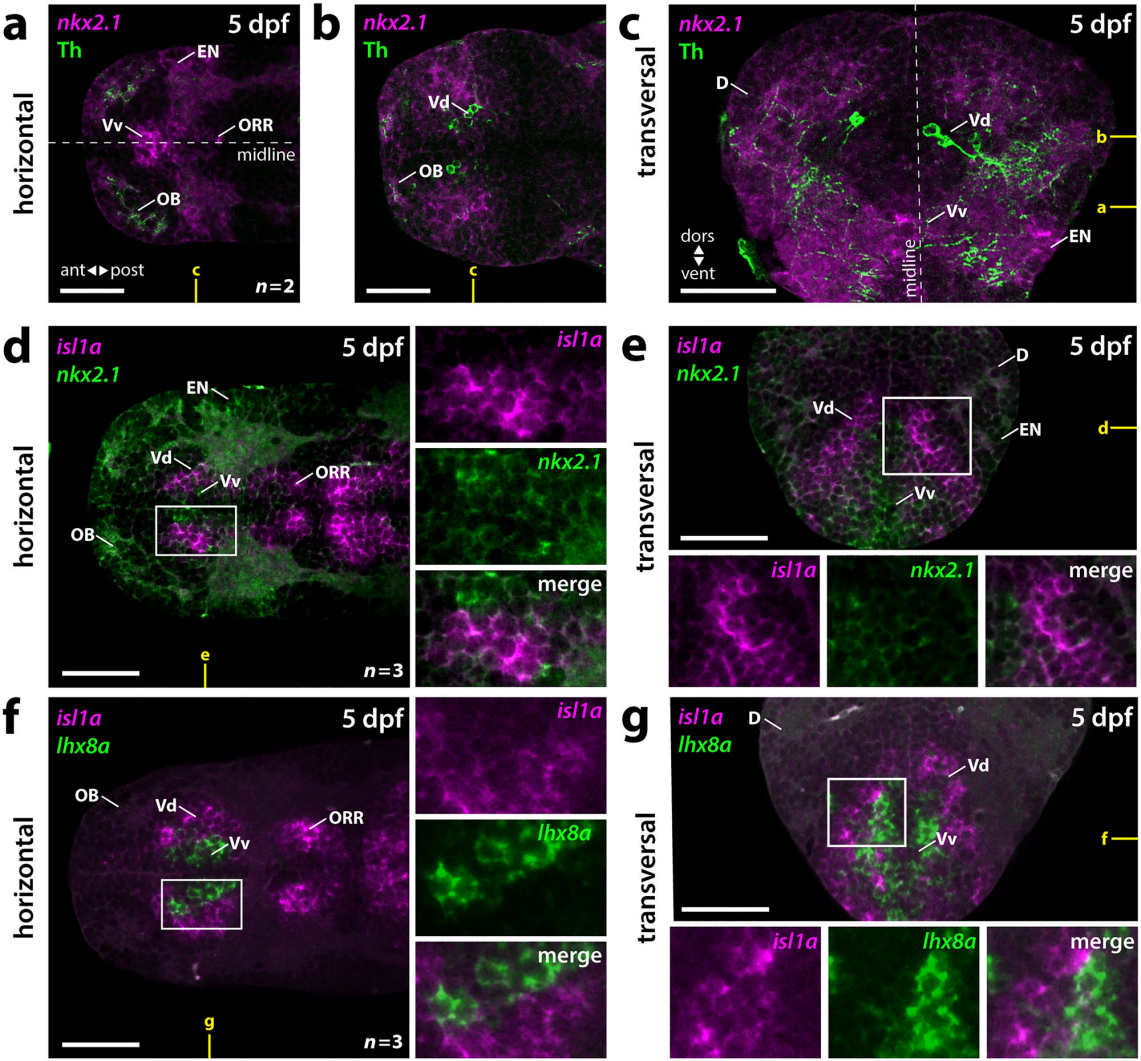
Subpallial DA neurons do not locate to MGE‐derived septopallidal regions. (a–c) Fluorescent in situ hybridization for *nkx2.1* that marks MGE derivatives in combination with Th immunostaining indicates that subpallial DA neurons are located dorsolateral of MGE‐derived septopallidal (Vv) regions. (a, b) Horizontal plains at the level of Vv (a) or Vdd (b) as marked in yellow in (c). (c) Transversal plane as marked in yellow in (a) and (b). (d, e) Double staining for *nkx2.1* and *isl1a* marks Vv and the striatal‐like Vd, respectively. (d) Horizontal plain at a level comprising Vv and Vd as depicted in yellow in (e). (e) Transversal plane at a precommissural level as marked in yellow in (d). (f, g) Double fluorescent in situ hybridization for *isl1a* and *lhx8a* validates the distinct localization of MGE‐derived Vv and vLGE‐derived Vd. (f) Horizontal plain at the level of Vv and Vd as marked in yellow in (g). (g) Transversal plane at a precommissural level as marked in yellow in (f). (a–g) White boxes mark the areas presented in the blowups. For better visualization, panels (a–e) were adjusted using nonlinear brightness and contrast enhancements. *n*, number of analyzed brains per experiment; ant, anterior; D, dorsal telencephalic area (pallium); dors, dorsal; dpf, days postfertilization; EN, entopeduncular nucleus; OB, olfactory bulb; ORR, optic recess region; post, posterior; Vd, dorsal division of the ventral telencephalon; Vdd, dorsalmost division of the ventral telencephalon; vent, ventral; Vv, ventral division of the ventral telencephalon. Scale bars = 50 µm.

To identify the exact localization and potential origin of the subpallial DA neurons, we performed coexpression studies of Th with transcription factors and differentiation markers that label distinct subpallial regions and lineages. We also analyzed the septopallidal region of Vv in order to translate our observations into a model of the larval zebrafish subpallium at 5 dpf (Figure 2f).

In mice, the transcription factor Nkx2.1 specifies MGE‐ and preoptic‐area‐derived telencephalic regions, such as the pallidum, septum, and EN (Sussel et al. 1999; Puelles et al. 2000; Xu et al. 2008). Likewise, in the zebrafish, telencephalon *nkx2.1* is expressed in the preoptic area as well as in pallidal and septal areas within Vv, including territories posterior to the anterior commissure referred to as the optic recess region (ORR) (Ganz et al. 2012; Manoli and Driever 2014; Affaticati et al. 2015). Subpallial DA neurons are neither located within *nkx2.1‐*positive Vv nor detected as coexpressing Th with *nkx2.1* (Figure 2a,b) (Filippi et al. 2012). The subpallial DA neurons are clearly located dorsolateral to *nkx2.1*‐expressing areas (Figure 2c). Dorsolateral to the *nkx2.1‐*positive regions in Vv, *isl1a* expression can be detected (Figure 2d,e). The *isl1a‐*positive regions likely correspond to striatopallidal structures of Vv and Vd (Stenman et al. 2003).

Another molecular marker for the MGE is Lhx8 (Grigoriou et al. 1998). Double staining of *isl1a* and *lhx8a* shows that MGE‐derived (*lhx8a‐*positive) Vv, and vLGE‐derived (*isl1a*‐positive) Vd are clearly distinct, with few coexpressing cells at the interface of both regions (Figure 2f,g). While *nkx2.1* expression can be detected already at and adjacent to the ventricle, *lhx8a* expression is absent from the ventricular zone, but present more laterally, potentially in differentiated neurons of Vv (see scheme in Figure 8). Together, these data demonstrate that the subpallial DA neurons are not located in MGE‐derived septopallidal regions of Vv.

### 3.3 Subpallial DA Neurons Are Located Dorsolateral of *isl1a*‐/*pax6a*‐Positive LGE Derivatives

Next, we asked in which anatomical subdivision of the dorsal subpallium, Vd(d) and more caudally Vs, the subpallial DA neurons are located. These regions contain the zebrafish homologs of mammalian vLGE (*isl1a*‐positive) and dLGE (*pax6*‐positive/*isl1a*‐negative) derived areas, including the proposed teleostean striatum Vd and parts of the subpallial amygdala in Vdd (Puelles et al. 2000; Tole et al. 2005; Bupesh et al. 2011; Ganz et al. 2012). Vdd of adult zebrafish has been described to be a largely *isl1a*‐free region containing the teleostean subpallial amygdala composed of the CeA, MeA, and BST homologs (Porter and Mueller 2020). In zebrafish, the main portion of telencephalic *pax6a‐*positive cells were described to be located in Vdd at the PSB, likely corresponding to the dLGE‐derived Pax6a‐positive cells in mammals that contribute to the striatum and CeA (Wullimann and Rink 2001; Bupesh et al. 2011). Thus, we suggest that also in the zebrafish Vdd, *pax6a* can serve as a marker for these dLGE derivatives and parts of the CeA.

**FIGURE 3:**
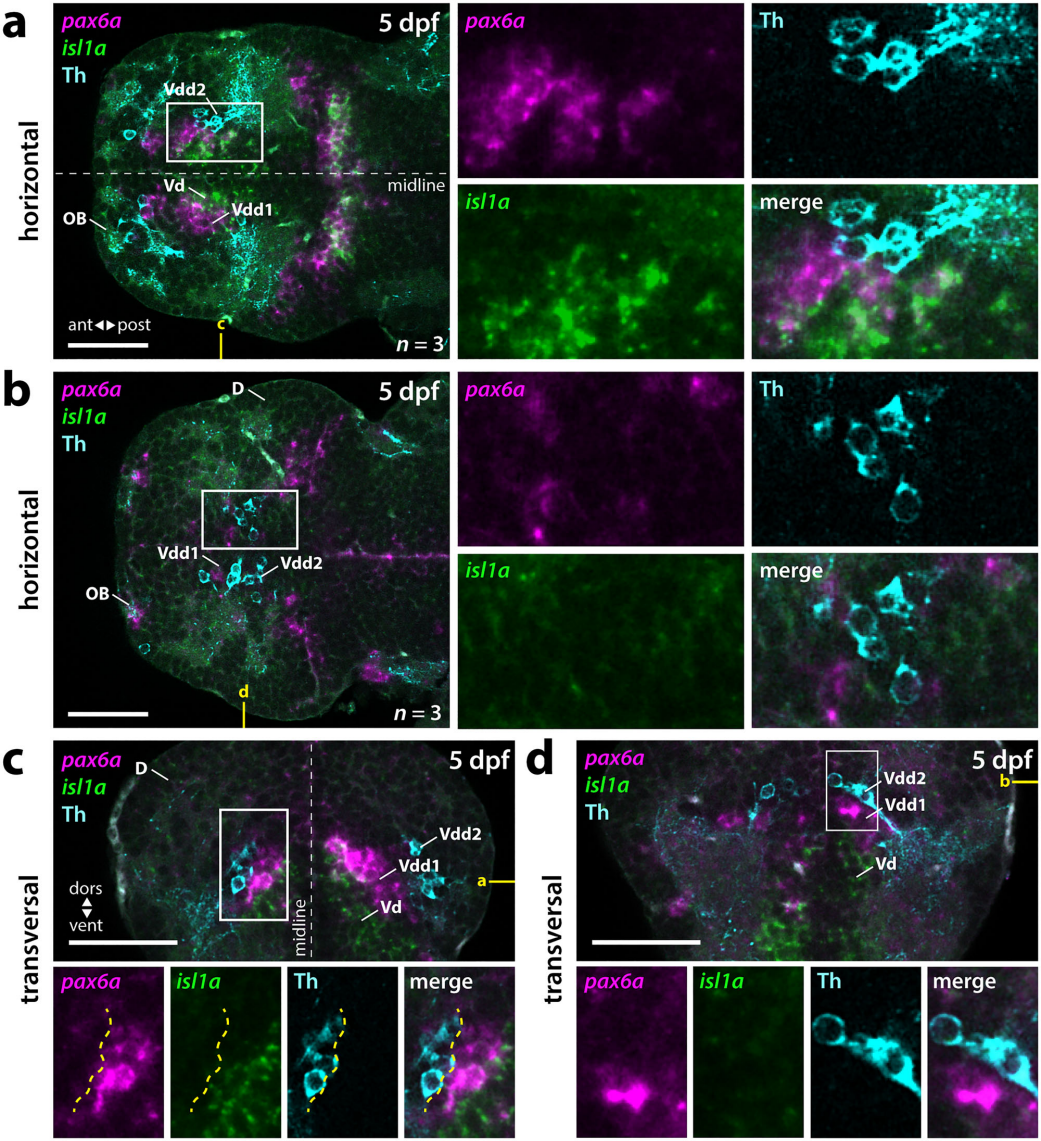
Subpallial DA neurons locate dorsolateral to the *isl1*‐expressing Vd and *pax6a*‐positive regions of Vdd1. (a–d) Double fluorescent in situ hybridization for *isl1a* and *pax6a* combined with Th immunofluorescence in 5‐dpf zebrafish brains. See also Video S1. (a, b) Horizontal planes at the level of Vd, Vdd1, and Vdd2 (a) and a more dorsal level containing Vdd1 and Vdd2 (b) as marked in yellow in (c) and (d). (c, d) Transversal planes at a precommissural level (c) and a supracommissural level (d) as marked in yellow in (a) and (b). Yellow dotted line in (c) marks the border of the *pax6a*‐expressing Vdd1 and of Vdd2. (a–d) White boxes mark the areas presented in the blowups. *n*, number of analyzed brains per experiment; ant, anterior; D, dorsal telencephalic area (pallium); dors, dorsal; dpf, days postfertilization; OB, olfactory bulb; post, posterior; Vd, dorsal division of the ventral telencephalon; Vdd1, dorsalmost division of the ventral telencephalon 1; Vdd2, dorsalmost division of the ventral telencephalon 2; vent, ventral. Scale bars = 50 µm.

We found that subpallial DA neurons are dorsolateral to *isl1a*‐positive Vd in 5‐dpf zebrafish (Figure 3a,c,d; Video S1). This dorsolateral positioning is more distinct rostral to the anterior commissure but still visible at supracommissural levels of Vs (Figure 3c,d). Therefore, the DA neurons are not located in the *isl1*‐positive Vd. We next asked whether we could use *pax6* expression as a marker to locate the DA neurons in specific Vdd anatomical entities.

Zebrafish have two *pax6* paralogs—*pax6a* and *pax6b* (Nornes et al. 1998); thus, due to subfunctionalization, *pax6b* may be expressed differentially from *pax6a*. To exclude the possibility that subpallial DA neurons may reside in a potentially more dorsal *pax6b* expression domain, we analyzed the expression of both *pax6* paralogs by WISH combined with Th immunofluorescence. We observed differential expression of both *pax6* paralogs in the 5‐dpf telencephalon. While *pax6a* is strongly expressed in the OB, *pax6b* is absent in this region (Figure 5c). In the subpallium, at more rostroventral positions near the OB, *pax6b* is predominantly expressed, while more caudally, *pax6a*‐ and *pax6b*‐double‐positive cells are located (Figure 5a–d). At supracommissural levels, however, only *pax6a* can be detected, while *pax6b* expression is largely absent (Figure 5e,h).

**FIGURE 4:**
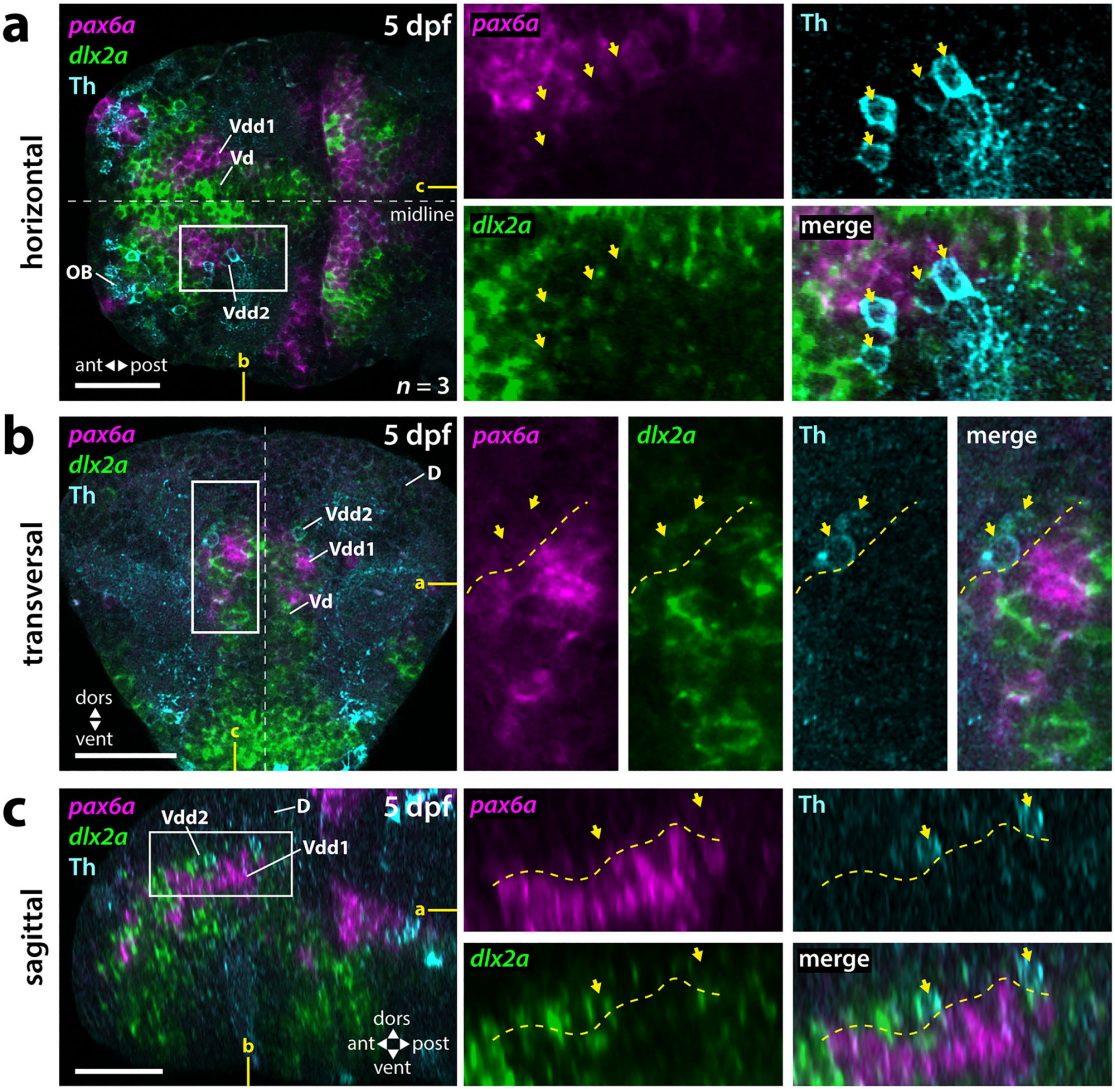
The subpallial DA neurons are located within an area of Vdd devoid of *pax6a* expression. (a–c) Double fluorescent in situ hybridization for *pax6a* and *dlx2a* combined with Th immunofluorescence in 5‐dpf brains. (a) Horizontal plane at the level of Vd, Vdd1, and Vdd2 as depicted in yellow in (b) and (c). (b) Transversal plane at a precommissural level as depicted in yellow in (a) and (c). (c) Sagittal plane through the entire telencephalon as depicted in yellow in (a) and (b). This plane derives from a computational reconstruction as described in the methods section. (a–c) White boxes mark the areas presented in the blowups. Yellow arrows mark Th‐immunoreactive cells. Yellow dotted line in (b) and (c) marks the border between the *pax6a*‐expressing Vdd1 and *pax6a*‐free Vdd2. *n*, number of analyzed brains per experiment; ant, anterior; D, dorsal telencephalic area (pallium); dors, dorsal; dpf, days postfertilization; OB, olfactory bulb; post, posterior; Vd, dorsal division of the ventral telencephalon; Vdd1, dorsalmost division of the ventral telencephalon 1; Vdd2, dorsalmost division of the ventral telencephalon 2; vent, ventral. Scale bars = 50 µm.

**FIGURE 5:**
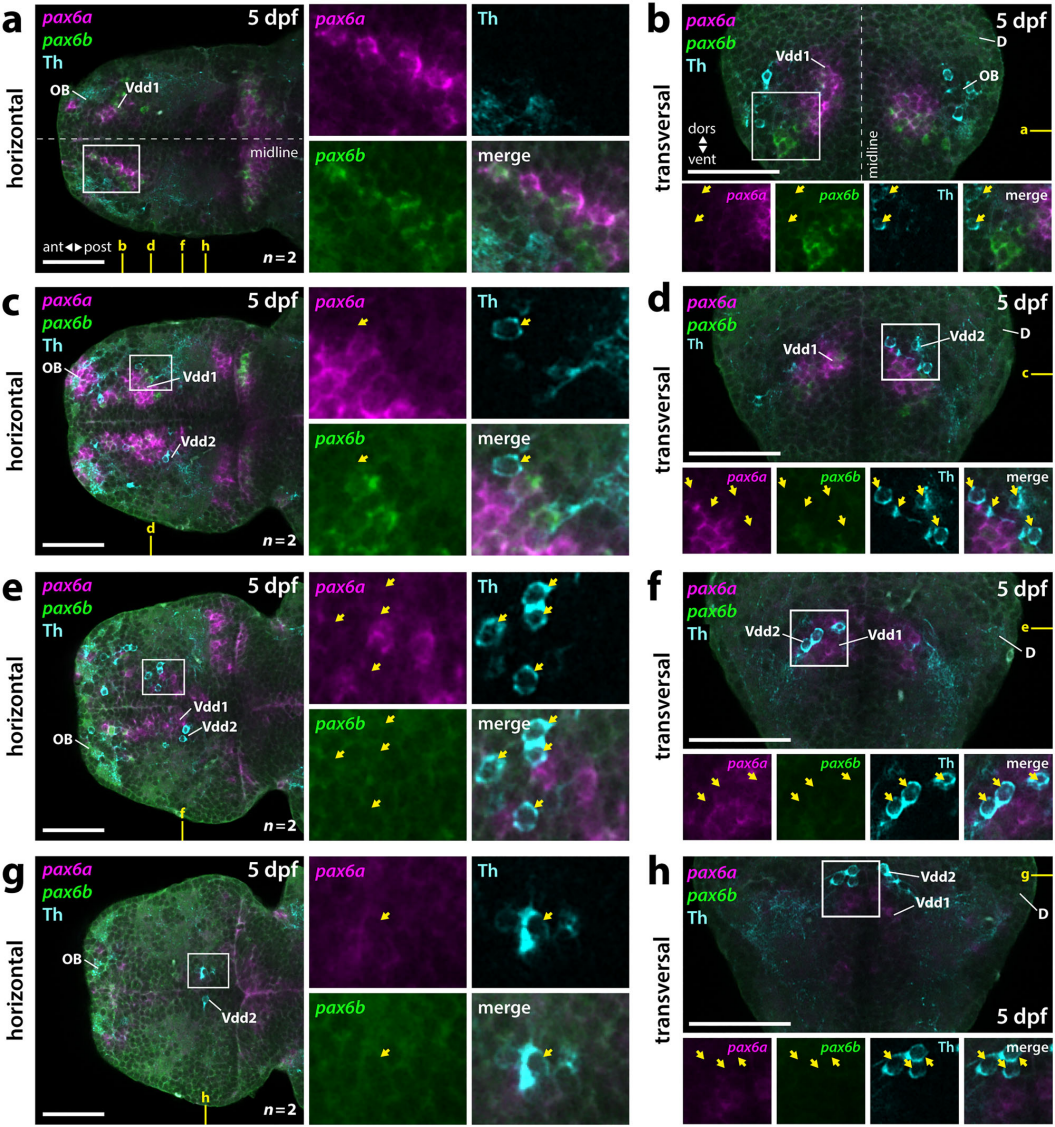
The *pax6a/b* are differentially expressed in the zebrafish telencephalon, but there is no co‐expression with Th. (a–h) Double fluorescent in situ hybridization for *pax6a* and *pax6b* combined with Th immunofluorescence in dissected brains. Panels (a), (c), (e), and (g) show progressively more dorsal horizontal planes, and panels (b), (d), (f), and (h) show progressively more caudal transversal sections (see yellow marks in [a]; corresponding horizontal and transversal images are indicated in each panel). (a, b) In the rostral subpallium, predominantly *pax6b* is expressed. (c, d) At more caudal positions, there is an intermingled expression of the two *pax6* paralogs. Yellow arrows mark Th‐immunoreactive neurons. (e–h) In the caudal subpallium and at supracommissural levels, only *pax6a* expression can be detected. White boxes mark the areas presented in the blowups. *n*, number of analyzed brains per experiment; ant, anterior; D, dorsal telencephalic area (pallium); dors, dorsal; dpf, days postfertilization; OB, olfactory bulb; post, posterior; Vdd1, dorsalmost division of the ventral telencephalon 1; Vdd2, dorsalmost division of the ventral telencephalon 2; vent, ventral. Scale bars = 50 µm.

The subpallial DA neurons locate very close and often directly adjacent to *pax6a*‐positive regions of Vdd (Figure 3b; Video S1). At supracommissural levels, individual DA neurons almost intermingle with *pax6a*‐positive cells (Figures 3b,c and 5e,f). However, the majority of the Th‐immunoreactive subpallial DA neurons are more dorsolateral to *pax6a*. This area represents a *pax6a‐*negative region of Vdd marked by the expression of *dlx2a* (Figure 4a–c). Also, for *pax6b*, we could detect individual intermingled *pax6b*‐ and Th‐immunoreactive cells (Figure 5c); however, as seen for *pax6b*, most of the Th‐immunoreactive cells are dorsolateral to the *pax6b*‐expressing cells (Figure 5d). Similar to *pax6a*, *pax6b* is not expressed in subpallial DA neurons. Thus, the subpallial DA neurons are located more dorsolateral to *pax6a/b* expressing regions of Vdd, which we term Vdd1, indicating the presence of a *pax6a*‐negative region of Vdd, termed here Vdd2. Vdd2 is *dlx2a* positive and is positioned directly adjacent to *tbr1b‐*positive pallial regions (Figure 8). Thus, the DA neuron containing region Vdd2 in the subpallium is located directly adjacent to the PSB.

### 3.4 *calb2a* Expression in a Subset of Subpallial DA Neurons Reveals Localization in the EMeA

The calcium‐binding protein Calretinin is expressed in several nuclei of the mammalian amygdala (Rowniak 2017). Within the subpallial amygdala, strong expression was detected in the medial nucleus. Also in zebrafish, the Calretinin homologs *calb2a/b* are expressed in the subpallium, including Vd, Vdd, Vs, and Vp (Castro et al. 2006). The expression of *calb2a/b* visualized by anti‐Calretinin immunoreactivity has also served as a marker for the EMeA, comprising the MeA and the BST in Vdd (Porter and Mueller 2020).

**FIGURE 6:**
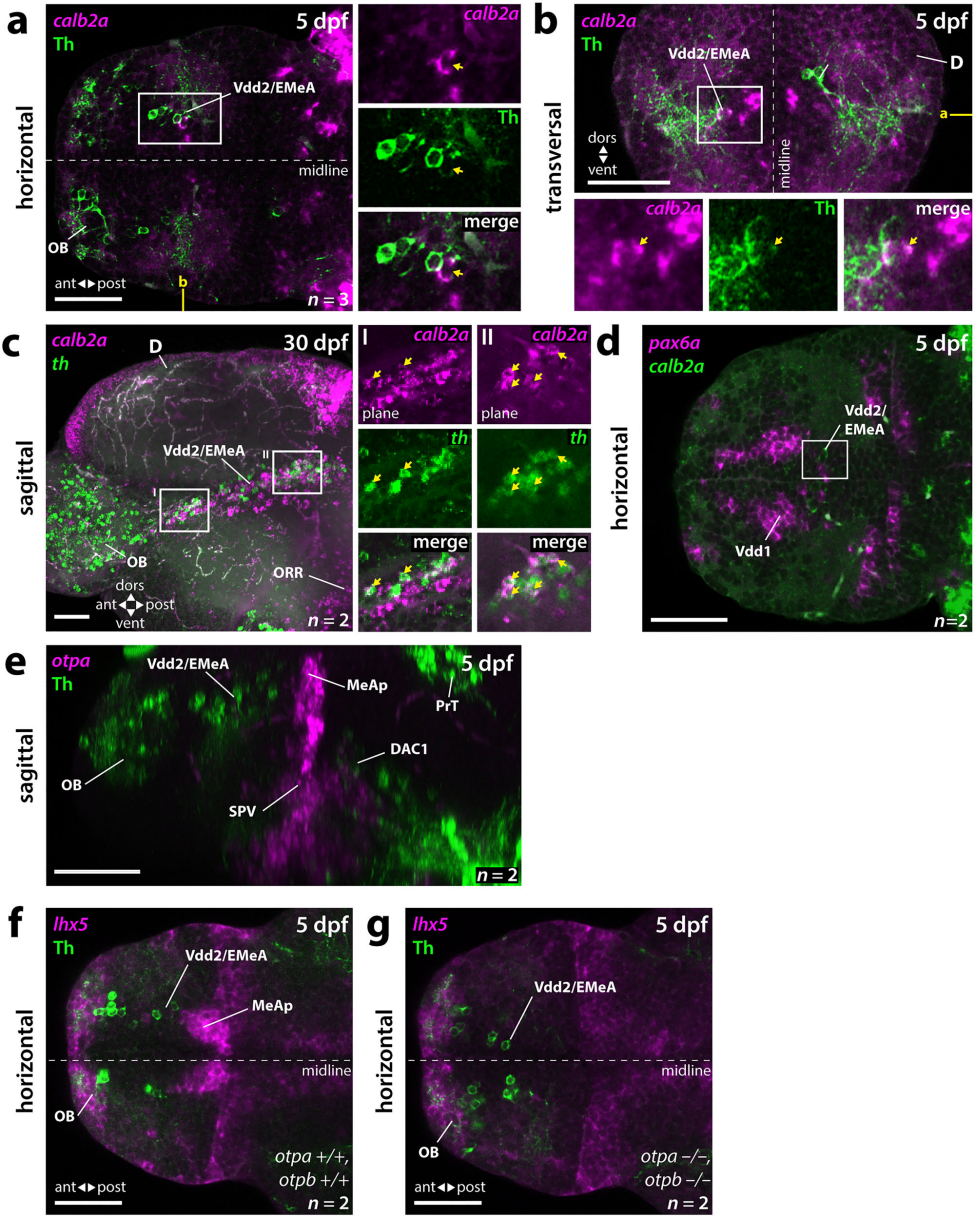
Calbindin expression localizes subpallial DA neurons to the extended medial amygdala within Vdd2. (a, b) WISH for *calb2a* in combination with Th immunofluorescence in 5‐dpf brains. Subpallial dopaminergic neurons are located within the EMeA domain of Vdd2 defined by *calb2a* expression. A subpopulation coexpresses *calb2a* and Th. See also Video S2. (a) Horizontal plain at the level of Vdd2/EMeA as marked in yellow in (b). (b) Transversal plane at a precommissural level marked in yellow in (a). (c) Sagittal *z*‐projection of an HCR for *calb2a* and *th* in 30‐dpf brains. In the 30‐dpf brain, *calb2a* is coexpressed in many *th*‐expressing neurons and *calb2a*‐positive cells intermingle with DA neurons along the entire rostrocaudal axis of the EMeA in Vdd2. The blowups represent the area marked by the white box as individual planes. (d) Double WISH for *calb2a* and *pax6a* in 5‐dpf brains*. calb2a‐*positive neurons do not coexpress *pax6a*. (e) WISH for *otpa* combined with Th immunofluorescence in 5‐dpf brains suggests that the Th cells of the Vdd2/EMeA do not contribute to the posterior MeA (MeAp) that is populated by *otpa*‐expressing cells. See also Video S3. (f, g) WISH for *lhx5* combined with Th immunofluorescence in 5‐dpf wildtype control (f) and *otpa/otpb* double mutants (g) shows that while the *lhx5‐*positive cells of the MeAp are absent, the Th‐expressing cells of Vdd2/EMeA develop normally. (a–d) White boxes mark the areas presented in the blowups. Coexpressing cells are marked by yellow arrows. *n*, number of analyzed brains per experiment; ant, anterior; D, dorsal telencephalic area (pallium); DAC1, dopaminergic cluster 1; dors, dorsal; dpf, days postfertilization; EMeA, extended medial amygdala; OB, olfactory bulb; ORR, optic recess region; MeAp, posterior medial amygdala; post, posterior; PrT, pretectum; SPV, supraoptoparaventricular area; sVdd1, dorsalmost division of the ventral telencephalon 1; Vdd2, dorsalmost division of the ventral telencephalon 2; vent, ventral. Scale bars = 50 µm.

We found that subpallial DA neurons in 5‐dpf zebrafish larvae are intermingled with a dorsal subpopulation of *calb2a‐*expressing cells in Vdd2. Indeed, a subpopulation of DA cells coexpresses Th and *calb2a* as judged from WISH (Figure 6a,b; Video S2). This observation could be extended to the 30‐dpf brain using HCR RNA detection, validating that at least a large subset of subpallial DA neurons is located in the *calb2a*‐positive Vdd2 (Figure 6c). A subset of DA neurons coexpress *calb2a* along the entire rostrocaudal extent of Vdd2 at 30 dpf (Figure 6c–e). We were not able to detect coexpression of *calb2a* and *pax6a* in the larval subpallium (Figure 6f). Thus, the zebrafish subpallial DA neurons are located in Vdd2, a Vdd territory devoid of *pax6a* expression and contributing to the EMeA.

### 3.5 Subpallial DA Neurons Do Not Coexpress *Otp* Paralogs

Recent work has demonstrated that in mice, neurons of the Otp lineage develop in the EMeA and show transitory TH expression (Bupesh et al. 2014; Morales et al. 2025). A telencephalic supraoptoparaventricular area (SPV) expresses *otp* and dorsocaudally extends to territories that contribute to components of the amygdala (Puelles and Rubenstein 2003). In zebrafish, this *otp*‐expressing cell population of the SPV is part of the ORR (Ryu et al. 2007; Affaticati et al. 2015). We analyzed the expression of the two paralogous *otpa* and *otpb* genes in relation to Th expression and detected no coexpression of *otp* and Th in the Vdd2/EMeA region (Figure 6e; Video S3). We reanalyzed archived data from our lab that document *th* expression in 3‐dpf *otpa otpb* double‐mutant larvae (Fernandes et al. 2013) and detected in four independent double mutants *th* expression in the subpallium similar to wildtype control larvae, indicating that at least by 3 dpf, *otp* activity is not required for the development of subpallial DA neurons. Therefore, we analyzed the expression of *lhx5* as a marker for the posterior medial amygdala (MeAp) (Porter and Mueller 2020) and TH in *otpa otpb* double mutants by combined WISH and immunofluorescence in 5‐dpf larvae. We found that while the MeAp domain of *lhx5* expression is affected in *otpa otpb* double mutants, Vdd2 DA neurons can still be detected (Figure 6f), demonstrating that Otp activity is indeed not required for larval DA neurons in Vdd2.

### 3.6 A Subset of Subpallial DA Neurons Expresses *sst7*

**FIGURE 7:**
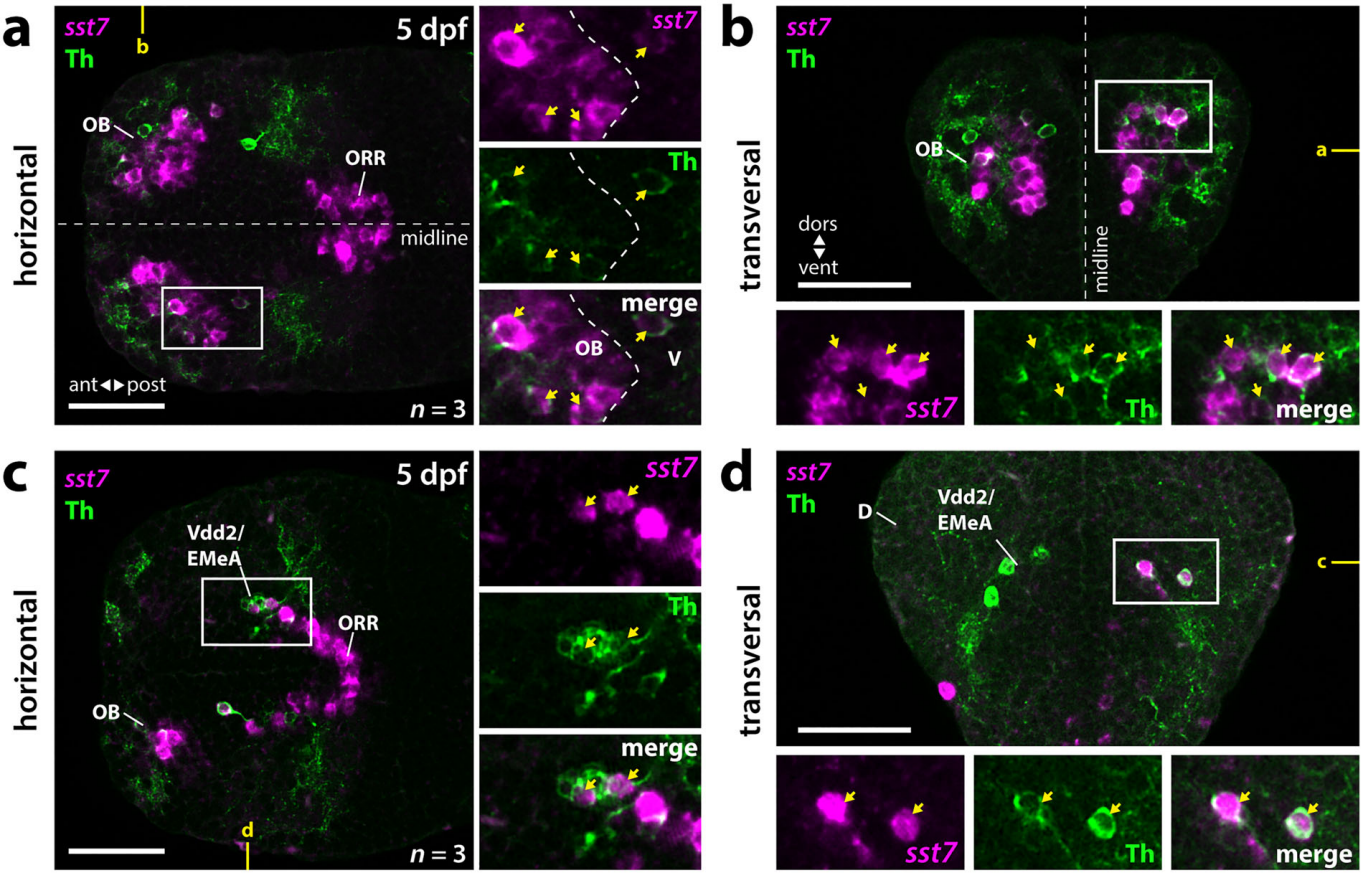
A subset of the EMeA DA neurons in Vdd2 express *somatostatin family member 7 (sst7)*. (a–d) WISH for *sst7* combined with anti‐Th immunofluorescence in 5‐dpf brains. (a, b) *sst7* is expressed within the olfactory bulb and optic recess region. A subset of olfactory bulb DA neurons coexpresses Th and *sst7*. See also Video S4. (a) Horizontal plane at a ventral level of V at the position marked in yellow in (b). Dotted line separates the olfactory bulb *sst7*‐expressing cells from the one in V. (b) Transversal plane at the level of the OB as marked in yellow in (a). (c, d) A subset of EMeA DA neurons in Vdd2 coexpresses *sst7*. (c) Horizontal plain at a more dorsal position containing Vdd2/EMeA as marked in yellow in (d). (d) Transversal plane at a precommissural level as marked in yellow in (c). (a–d) White boxes mark the areas presented in the blowups. Coexpressing cells are marked by yellow arrows. *n*, number of analyzed brains per experiment; ant, anterior; D, dorsal telencephalic area (pallium); dors, dorsal; dpf, days postfertilization; EMeA, extended medial amygdala; OB, olfactory bulb; ORR, optic recess region; post, posterior; V, ventral telencephalic area (subpallium); Vdd2, dorsalmost division of the ventral telencephalon 2; vent, ventral. Scale bars = 50 µm.

In mammals, the neuropeptide Cortistatin is expressed, among other anatomical regions, in the basolateral amygdala, the striatum, and the medial amygdala (de Lecea, Ruiz‐Lozano, et al. 1997). So far, the telencephalic distribution of the zebrafish Cortistatin ortholog Sst7 (*somatostatin family member 7*) has not been described in detail (Tostivint et al. 2004). We detected *sst7* expression in the OB, the subpallium, and, caudal to the anterior commissure, the ORR (Figure 7; Video S4). In the subpallium, *sst7* expression is restricted to the region in Vdd2 that we consider to contain the EMeA in 5‐dpf zebrafish. In fact, a subset of subpallial DA neurons also expresses *sst7* (Figure 7). Thus, some subpallial DA neurons in Vdd2/EMeA likely produce the neuropeptide Sst7.

## 4 Discussion

The absence of mesodiencephalic DA systems in zebrafish raises the question whether there are any DA systems that provide substantial DA input into the striatum, limbic systems, and pallium (Smeets and Gonzalez 2000; Yamamoto and Vernier 2011). A potential source for dopamine is the subpallial DA group in zebrafish (Holzschuh et al. 2001; Kaslin and Panula 2001; Filippi et al. 2010; Yamamoto et al. 2010); however, the exact anatomical location, development, and function of these neurons have not been well understood to this day. Here, we aim to identify the subpallial subdivisions in which the DA neurons reside and define new markers to better resolve their identity. We focus our analysis on the 5‐dpf early larval brain, when all major telencephalic subdivisions have established (Mueller and Wullimann 2016), and the brain is still small enough such that the telencephalon can be documented entirely in a single confocal stack. We also analyze 30‐dpf brains when the adult brain morphology has been established. Combined analyses of Th immunolocalization for DA neurons, *lhx8*, *dlx2a*, *nkx2.1*, *isl1a*, *pax6a*, and *tbr1b* as anatomical markers, as well as of *gad1a/1b/2*, *calb2a*, and *sst7* as differentiation markers demonstrate that the zebrafish subpallial DA neurons are located in the EMeA and, with respect to *calb2a* and *sst7*, constitute a diverse group of neurons.

### 4.1 Subdivisions of the Larval Zebrafish Subpallium at 5 dpf

**FIGURE 8:**
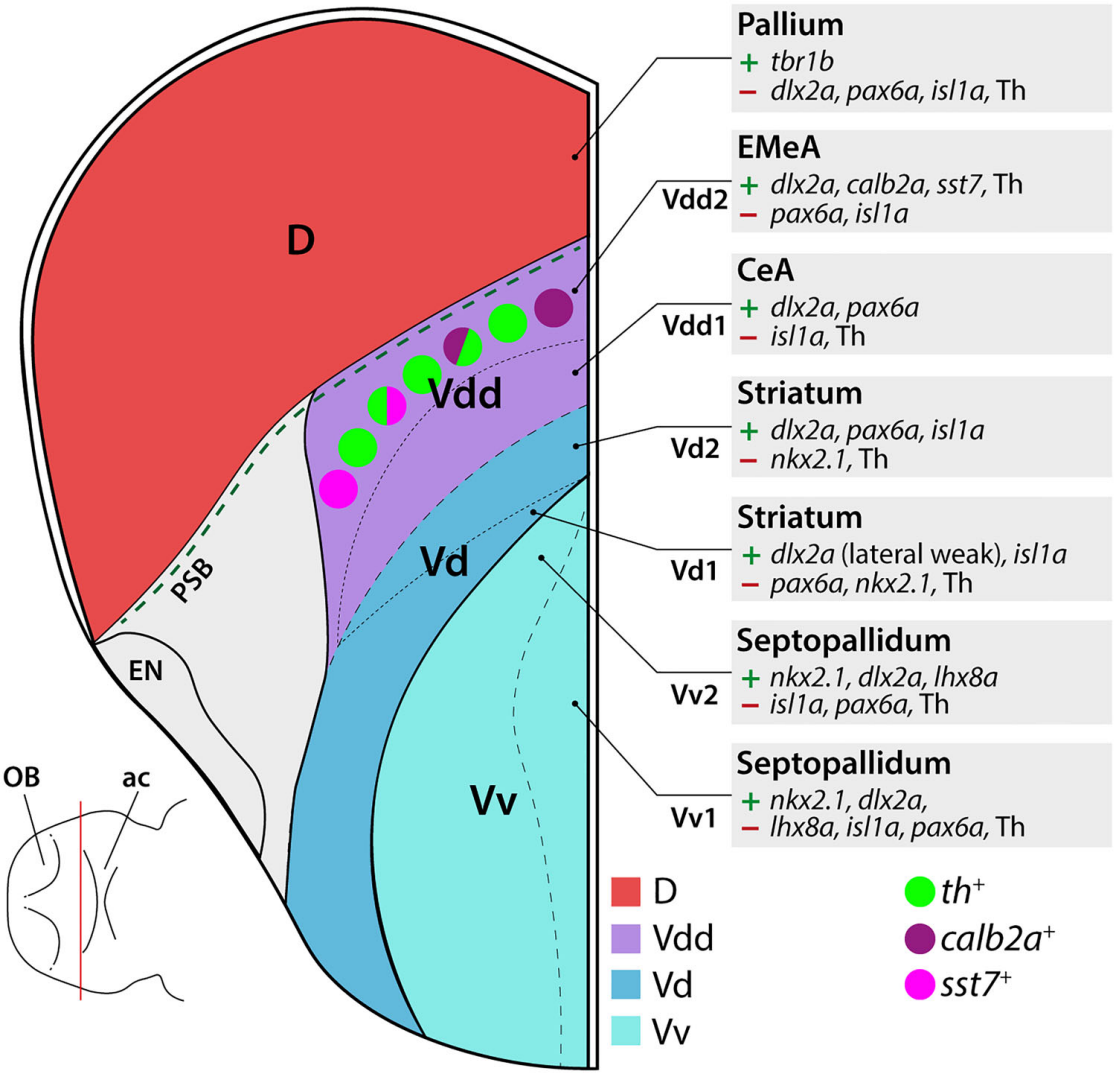
Schematic representation of localization of DA neurons relative to telencephalic divisions analyzed in this study. DA neuron subpopulations expressing *calb2a* or *sst7* are indicated. Transversal representation of one hemisphere of the 5‐dpf telencephalon. ac, anterior commissure; CeA, central amygdala; D, dorsal telencephalic area; EMeA, extended medial amygdala; EN, entopeduncular nucleus; OB, olfactory bulb; PSB, pallial‐subpallial border; V, ventral telencephalic area; Vd, dorsal division of the ventral telencephalon; Vdd, dorsalmost division of the ventral telencephalon; Vv, ventral division of the ventral telencephalon.

Previous studies on the subpallial topology of adult zebrafish defined homologies to MGE‐ and vLGE/dLGE‐derived structures such as pallidal, striatal, and amygdaloid nuclei (Porter and Mueller 2020). To understand their development, a more profound knowledge about the telencephalic anatomy at early stages is needed. Our analyses of *dlx2a*, *isl1a*, *lhx8*, *nkx2.1*, *pax6a*, and *tbr1b* expression in the telencephalon validate existing subdivisions and suggest additional ones, such that we propose six subdivisions of the ventral telencephalon rostral to the anterior commissure (Figure 8): two *nkx2.1‐*positive septopallidal domains Vv (correlating to MGE), distinguished by *lhx8a* expression which can only be detected in Vv2 but is absent from Vv1; two ventral subdomains (correlating to ventral LGE), a striatal Vd1 with *isl1* expression and a Vd2 with both *isl1* and *pax6* expression; and two Vdd subdivisions (correlating to dorsal LGE), including the more ventral Vdd1 of CeA character with *dlx2a* and *pax6a* expression, but no *isl1*, and the Vdd2 of EMeA character, which is *pax6a* negative but contains *calb2a‐* and sst7‐positive cells.

These markers have been shown to identify ventral telencephalic subdivisions throughout vertebrate evolution. The knockout of the transcription factor Nkx2.1 in mice results in the loss of MGE and an expansion of LGE‐derived tissues (Sussel et al. 1999), and knockdown in *Xenopus* and zebrafish reveals an important role in controlling the relative sizes of major regions of both telencephalon and diencephalon (van den Akker et al. 2008; Manoli and Driever 2014), together identifying a highly conserved Nkx2.1 function in dorsoventral forebrain patterning (Puelles et al. 2000; Moreno 2007). *Lhx8* is required for the specification of cholinergic cell types of the MGE lineage that reside within the pallidum (Zhao et al. 2003). The more dorsolaterally located *isl1a*‐positive and *pax6a/lhx8a*‐negative region resembles the *isl1a‐*positive vLGE, which gives rise to GABAergic projection neurons of the teleostean striatum and has already been described for zebrafish and many other vertebrates, including rodents, amphibians, and birds (Stenman et al. 2003; Moreno et al. 2008; Abellán and Medina 2009; Ganz et al. 2012). The dorsalmost part of the zebrafish subpallium Vdd was described as *isl1a*‐free (Ganz et al. 2012; Porter and Mueller 2020). The *isl1a*‐free Vdd expresses the transcription factor *pax6a*, likely resembling the Pax6‐positive dLGE whose derivatives constitute parts of the CeA in tetrapods and also form part of the BST in mice (Yun et al. 2001; Abellán and Medina 2009; Moreno et al. 2010; Bupesh et al. 2011). Thus, we suggest that the *pax6a‐*positive Vdd1 domain partially contributes to developing CeA‐like structures of larval zebrafish. This is in line with the description of the subpallial amygdala of adult zebrafish as a largely *isl1a*‐free GABAergic region ventral to the pallium (Porter and Mueller 2020). Dorsolateral to the *pax6a‐*positive Vdd1 domain, we could detect scattered cells in Vdd2 expressing *calb2a*, which were described to form part of the teleostean equivalents of the EMeA in the adult brain (Porter and Mueller 2020). In the same region, we could observe numerous *sst7* neurons.

Thus, the subpallium of 5‐dpf larval zebrafish already exhibits striking similarities to the subpallium of adult zebrafish, with a high degree of regional identity and conservation of subpallial subdivisions of tetrapods. From an evolutionary perspective, our results support the notion that a tetrapod‐like prosomerically organized amygdaloid complex originated in early vertebrates in a common ancestor of ray‐finned fish and tetrapods (Porter and Mueller 2020; Wullimann 2022). The largely conserved topology of the zebrafish amygdala, as exemplified in this study for a portion of the subpallial EA, is the result of genetic and ontogenetic constraints shared between ray‐finned fish and tetrapods.

### 4.2 Teleostean Subpallial DA Neurons Are Located Within the EMeA

Considering that zebrafish subpallial DA neurons strongly innervate the telencephalon (Tay et al. 2011), it is intriguing to consider that zebrafish subpallial DA neurons may have similar neuromodulatory functions in the telencephalon as midbrain DA neurons of mammals. To test the hypothesis of the amygdaloid localization of the subpallial DA neurons (Porter and Mueller 2020), we analyzed the expression of marker genes suitable for defining subpallial regions in combination with the distribution of Th immunoreactivity. The GABAergic subpallial DA neurons in 5‐dpf zebrafish reside at the PSB medioventrally adjacent to *tbr1b‐*positive pallial regions. This *dlx2a*‐positive region of Vdd, Vp, and Vs was described as the teleostean subpallial amygdala in adult zebrafish (Mueller et al. 2008; Ganz et al. 2012; Porter and Mueller 2020). Porter and Mueller (2020) locate subpallial DA neurons in the adult EMeA based on their position dorsolateral to the *isl1a‐*positive Vd, which corresponds to vLGE‐derived striatal regions of mammals. Our finding that already in the larval brain the subpallial DA neurons are located dorsolateral to the *isl1a* expression domain corroborates their amygdaloid nature (Vdd). Interestingly, our data point to a novel early subdivision in Vdd. Previously, the *pax6a* expression domain was considered to form the PSB in zebrafish (Wullimann and Rink 2002). Our findings, however, suggest that a *dlx2a‐*positive and *pax6a‐*negative region of the dLGE‐like Vdd forms at the PSB that we term Vdd2. We locate the subpallial DA neurons exactly in this *pax6a*‐ and *isl1*‐negative Vdd2 area.

Recently, lineage analysis in mice revealed that some TH‐expressing cells in the posterior medial EA derive from a forebrain Otp‐expressing lineage of the SPV (Morales et al. 2025). In our study, we failed to detect coexpression of the two paralogous *otpa* and *otpb* genes in Vdd2 DA neurons. Given that migrating SPV‐derived *otp*‐expressing progenitors might turn *otp* expression off before differentiating into DA neurons, we cannot exclude an SPV contribution to Vdd2 EMeA DA neurons. However, our analyses of *otpa otpb* double mutants revealed that at least the early developing subpallial DA population by 5 dpf does not depend on *otp* activity. Both based on anatomical location and lineage, we do not consider the Vdd2 DA neurons homologous to the mammalian DA neurons of the Otp‐positive lineage migrating to the posterior EMeA.

The Vdd2 area to which the subpallial DA neurons are located is characterized by dispersed *calb2a* (Calretinin homolog)‐expressing neurons, and indeed some of the DA neurons coexpress *calb2a*. In mammals, Calbindin‐ and Calretinin‐expressing neurons populate major parts of various amygdaloid nuclei—including the EMeA (Wójcik et al. 2013; Rowniak 2017). Likewise, in adult zebrafish, anti‐Calretinin immunoreactivity marks the EMeA, including the BST (Porter and Mueller 2020). Thus, the presence of *calb2*‐expressing GABAergic neurons in Vdd2 corroborates the extended medial amygdaloid nature of these territories. Among others, the MeA is implicated in mediating fear responses as well as in the regulation of social behavior by receiving olfactory and vomeronasal inputs (Meredith et al. 1999; Herdade et al. 2006).

A further indication of subtype diversity in the EMeA DA population is provided by the expression of *sst7*. *sst7* codes for the somatostatin family member Sst7, an ortholog to the mammalian neuropeptide Cortistatin. Cortistatin expression can be detected in GABAergic interneurons of the cortex as well as in the hippocampus and in neurons of the OB, the basolateral amygdala, the striatum, the BST, and the MeA (de Lecea et al. 1996; de Lecea, Del Rio, et al. 1997; de Lecea, Ruiz‐Lozano, et al. 1997; Tallent et al. 2005). Similarly, in the marsh frog (*Rana ridibunda*), the suggested Cort ortholog PSS2 is expressed in the medial pallium, but also in the preoptic area and the MeA (Tostivint et al. 1996). Our finding of *sst7* expression in 5‐dpf zebrafish larvae EMeA and DA neurons indicates strong conservation.

### 4.3 Conclusions and Further Perspective

Our study advances two major aspects of the teleostean subpallial organization and function in zebrafish. First, we refine the anatomical organization of the dorsal LGE‐like Vdd into a Vdd2 subdivision that we propose corresponds to the EMeA and forms an additional part of the PSB region, and a more ventral Vdd1 that is *pax6a* positive and may correspond to the CeA. Second, we define the subpallial DA neurons as a part of the EMeA in larvae and adult zebrafish. The presence of TH‐immunoreactive neurons in the EA of the adult prairie vole, as well as transient *TH*‐expressing populations in the central and medial amygdala of mouse embryos, indicates an early evolutionary origin of amygdaloid DA neurons (Northcutt et al. 2007; Bupesh et al. 2014). Our findings shed light on potential mechanisms of DA system evolution. From an ancestral simple form that contained only a few subpallial and mesodiencephalic DA systems, mammals evolved the more complex mesolimbic, mesostriatal, and mesocortical systems that serve the needs of neuromodulation of complex behaviors. The zebrafish subpallial DA neurons may also provide an interesting model to investigate local DA differentiation and establishment of connectivity within the subpallium and wider forebrain. A better understanding of this system may have a strong potential to understand and model amygdala and dopamine‐related disorders, including addiction, depression, schizophrenia, and social behavior deficits (Schultz 2002; Grace 2016; Opendak et al. 2021).

## Author Contributions

Daniel Armbruster designed the study, performed all experiments, compiled the data, conducted formal analysis, generated visualizations, assembled figures, wrote the original draft, and edited manuscript drafts. Thomas Mueller conceived and designed the study, provided supervision, and reviewed and edited manuscript drafts. Wolfgang Driever conceived and designed the study, provided resources, supervision, and funding acquisition, and contributed to writing, reviewing, and editing of the manuscript.

## Conflicts of Interest

The authors declare no conflicts of interest.

## Peer Review

The peer review history for this article is available at https://publons.com/publon/10.1002/cne.70079

## Supporting information




**Supplementary Materials**: cne70079‐sup‐0001‐SuppMat.docx

**Supplementary Video**: cne70079‐vid‐0002‐Video1.mp4

**Supplementary Video**: cne70079‐vid‐0003‐Video2.mp4

**Supplementary Video**: cne70079‐vid‐0004‐Video3.mp4

**Supplementary Video**: cne70079‐vid‐0005‐Video4.mp4

## Data Availability

The data that support the findings of this study are available from the corresponding author upon reasonable request.
