# Supplementary material for: Dopaminergic Neurons in the Zebrafish Subpallium Belong to the Extended Medial Amygdala
Source: J Comp Neurol. 2025 Aug 5;533(8):e70079. doi: 10.1002/cne.70079 (PMC12324066; doi:10.1002/cne.70079)
Supplement: Supplementary file 1 — Supplementary Materials: cne70079‐sup‐0001‐SuppMat.docx [file CNE-533-e70079-s002.docx]

**Dopaminergic Neurons in the Zebrafish Subpallium Belong to the Extended Medial Amygdala**

Daniel Armbruster, Thomas Mueller, and Wolfgang Driever

**Supplementary Materials**

**Supplementary Video Legends (page 1)**

**Supplementary Tables**

Table S1: Probes for WISH used in this study (page 2)

Table S2: HCR Probes used in this study (page 3)

**Supplementary References (page 4)**

**Supplementary Videos S1, S2, S3 and S4**

**Supplementary Video 1** (related to Figure 3) Subpallial DA neurons locate dorsolateral to both vLGE and the pax6 positive regions of dLGE. Double fluorescent *in-situ* hybridization for *isl1a* and *pax6a* combined with Th immunofluorescence in 5 dpf zebrafish brains. (Format MP4 1920 x 1080 pixel, 20.2 Mb).

**Supplementary Video 2** (related to Figure 6) Calbindin expression localizes subpallial DA neurons to the medial extended amygdala. WISH for *calb2a* in combination with Th immunofluorescence in 5 dpf brains. (Subpallial dopaminergic neurons are located within a domain of the EMeA defined by *calb2a* expression. A subpopulation coexpresses *calb2a* and Th. (Format MP4 1920 x 1080 pixel, 14.5 Mb).

**Supplementary Video 3** (related to Figure 6) The Vdd2/EMeA DA neurons are located anterior to the *otpa* and *otpb* expressing pMeA and do not coexpress the *otp* paralogues. WISH for *otpa* and *otpb* in combination with Th immunofluorescence in 5 dpf brains. The red asterisk shown between z86 and z96 marks an endothelial cell of a brain blood vessel (Format MP4 1920 x 1080 pixel, 30.0 Mb).

**Supplementary Video 4** (related to Figure 7) A subset of subpallial DA neurons express *somatostatin family member 7* *(sst7)*. WISH for *sst7* combined with anti-Th immunofluorescence in 5 dpf brains. *sst7* is expressed within the olfactory bulb and optic recess region. A subset of olfactory bulb DA neurons coexpresses Th and *sst7.* (Format MP4 1920 x 1080 pixel, 12.7 Mb).

Table S1: Probes for WISH used in this study

| cDNA | Ensembl Transcript ID | Vector | Linearization Enzyme | RNA Polymerase | Reference |
| --- | --- | --- | --- | --- | --- |
| *dlx2a* | ENSDART00000111386.4 | pBluescript | BamHI | T7 | (Akimenko et al., 1994) |
| *gad1a* | ENSDART00000025332.9 | pBluescript SK- | EcoRI | T3 | (Martin et al., 1998) |
| *gad1b* | ENSDART00000122348.4 | pBluescript SK- | EcoRI | T3 | (Martin et al., 1998) |
| *gad2* | ENSDART00000021609.10 | pBluescript SK- | EcoRI | T3 | (Martin et al., 1998) |
| *lhx5* | ENSDART00000080693.5 | pBlueskript | BamHI | T7 | (Toyama et al., 1995) |
| *isl1a* | ENSDART00000010896.6 | pBluescript KS+ | XbaI | T3 | (Tokumoto et al., 1995) |
| *nkx2.1* | ENSDART00000017493.7 | pSportI | KpnI | SP6 | (Manoli & Driever, 2014) |
| *otpa* | ENSDARG00000014201) | TopoPCRII | XhoI | Sp6 | (Ryu et al., 2007) |
| *otpb* | ENSDARG00000058379 | TopoPCRII | KpnI | T7 | (Ryu et al., 2007) |
| *pax6a* | ENSDART00000164384.2 | pGEM-3 zf (+) | BamHI | T7 | (Krauss et al., 1991) |
| *pax6b* | ENSDART00000192252.1 | pBluescript | EcoRI | T7 | (Nornes et al., 1998) |
| *tbr1b* | ENSDART00000006612.7 | pCR2-TOPO | BamHI | T7 | This study |
| *calb2a* | ENSDART00000060160.5 | pCR2-TOPO | XhoI | SP6 | This study |
| *cort/sst7* | ENSDART00000047378.7 | pCR2-TOPO | XhoI | SP6 | This study |
| *lhx8a* | ENSDART00000019078.5 | pCR2-TOPO | EcoRV | SP6 | This study |

**Table S2: HCR Probes used in this study.**

| **Name** | **5’ to 3’ sequence** | **Reference** |
| --- | --- | --- |
| calb2a_HCR_Oligo1.1 | GTCCCTGCCTCTATATCTTTGCCATCTTATCGTATACTGTCCACA | this study |
| calb2a_HCR_Oligo1.2 | AGGTAAGGAGGCTGCTGTCCTTTGTTTCCACTCAACTTTAACCCG |  |
| calb2a_HCR_Oligo2.1 | GTCCCTGCCTCTATATCTTTAGCTCCTTACCCTCGATGTAGCCAT |  |
| calb2a_HCR_Oligo2.2 | ATCTCCAGCTGACTAAAGAAGTTGTTTCCACTCAACTTTAACCCG |  |
| calb2a_HCR_Oligo3.1 | GTCCCTGCCTCTATATCTTTTCTCCCCTTAAAGGCTGCATTTGAG |  |
| calb2a_HCR_Oligo3.2 | GTCGAATTTCTGCATGAACTCTGTCTTCCACTCAACTTTAACCCG |  |
| calb2a_HCR_Oligo4.1 | GTCCCTGCCTCTATATCTTTTCCCACAAACTGTCTGAAGCAGAGG |  |
| calb2a_HCR_Oligo4.2 | CCAGGCAGCCATGAATTCGGTGCTGTTCCACTCAACTTTAACCCG |  |
| calb2a_HCR_Oligo5.1 | GTCCCTGCCTCTATATCTTTTGCCTTTTTAAGCAGGTCAGAGAGG |  |
| calb2a_HCR_Oligo5.2 | CAATTTCTGGTCGTCGTAATGTCTGTTCCACTCAACTTTAACCCG |  |
| calb2a_HCR_Oligo6.1 | GTCCCTGCCTCTATATCTTTCAGAAGCCTTGCCATTTCAGACAGA |  |
| calb2a_HCR_Oligo6.2 | CTTCAACAAGAAGTTCTCCTGAACTTTCCACTCAACTTTAACCCG |  |
| calb2a_HCR_Oligo7.1 | GTCCCTGCCTCTATATCTTTGCTCGTCAATGTAGCCGTTTCCATC |  |
| calb2a_HCR_Oligo7.2 | GGTCCTTCAGGAGAGCGTCCAACTCTTCCACTCAACTTTAACCCG |  |
| calb2a_HCR_Oligo8.1 | GTCCCTGCCTCTATATCTTTTTGCCTCCATCCGACAGGGCCATGA |  |
| calb2a_HCR_Oligo8.2 | ACAATCTCCAGCTCTGAGCGGTACATTCCACTCAACTTTAACCCG |  |
| calb2a_HCR_Oligo9.l | GTCCCTGCCTCTATATCTTTGCCCGGCACATGCACACGGTTACAG |  |
| calb2a_HCR_Oligo9.2 | GGGGTGGGGACGGGATGGGGACGGGTTCCACTCAACTTTAACCCG |  |
| calb2a_HCR_Oligo10.1 | GTCCCTGCCTCTATATCTTTCAGTGGTGATTGGGTTTTGGGCAGG |  |
| calb2a_HCR_Oligo10.2 | TAGCTGTAGTTTGTAGGCAGAGGAATTCCACTCAACTTTAACCCG |  |
| HCR_Dan_th_B4_1 | CCTCAACCTACCTCCAACAAAAGATGTGGAGCTGCTTGAATTCGG | Altbürger, 2023 |
| HCR_Dan_th_B4_2 | ACGCAGCTCTCCGGATGCTCTTTGTATTCTCACCATATTCGCTTC |  |
| HCR_Dan_th_B4_3 | CCTCAACCTACCTCCAACAACCAACAAACTTCTGAGACGTAATAG |  |
| HCR_Dan_th_B4_4 | GCATCCTCGATCAAACTCTGCCGCCATTCTCACCATATTCGCTTC |  |
| HCR_Dan_th_B4_5 | CCTCAACCTACCTCCAACAAGCTGGGTCTGGTTTCAAGATGGTGG |  |
| HCR_Dan_th_B4_6 | GTCCTCCAAGCCATCCTTTGGTTTTATTCTCACCATATTCGCTTC |  |
| HCR_Dan_th_B4_7 | CCTCAACCTACCTCCAACAAAACATCCGACAGGTGCACTTCACAC |  |
| HCR_Dan_th_B4_8 | TCTCTTCAAAGAGCTGACCAGCGTGATTCTCACCATATTCGCTTC |  |
| HCR_Dan_th_B4_9 | CCTCAACCTACCTCCAACAAGGTGATCCTGATCCAGATCCGGATC |  |
| HCR_Dan_th_B4_10 | TTCTGTAAACAGGGTCAGTAAATCCATTCTCACCATATTCGCTTC |  |
| HCR_Dan_th_B4_11 | CCTCAACCTACCTCCAACAATCTGTATTTGAAGGCAATGTCTCCG |  |
| HCR_Dan_th_B4_12 | GTCCACTCTAGGAATTGGTTCTCCAATTCTCACCATATTCGCTTC |  |
| HCR_Dan_th_B4_13 | CCTCAACCTACCTCCAACAAAGCCGCAATGTTTCTCCAGTAACCG |  |
| HCR_Dan_th_B4_14 | CCAGCTGAGGGATGTTATCTGGGCTATTCTCACCATATTCGCTTC |  |
| HCR_Dan_th_B4_15 | CCTCAACCTACCTCCAACAACACTGGCCTCAACTGAAATCCTGTG |  |
| HCR_Dan_th_B4_16 | AAAATCGCGTGCTGAGAGCAAACCTATTCTCACCATATTCGCTTC |  |
| HCR_Dan_th_B4_17 | CCTCAACCTACCTCCAACAAAGTGAACCAGTACATTGTCGATAGC |  |
| HCR_Dan_th_B4_18 | TCCCTGTTTACACAGTCCAAACTCCATTCTCACCATATTCGCTTC |  |
| HCR_Dan_th_B4_19 | CCTCAACCTACCTCCAACAAGGTAAGTCTGGTCTTGGTATGGCTG |  |
| HCR_Dan_th_B4_20 | AGCTTTCTGACACAAAATAGACAGGATTCTCACCATATTCGCTTC |  |

**Supplementary References**

Akimenko, M., Ekker, M., Wegner, J., Lin, W., & Westerfield, M. (1994). Combinatorial Expression of Three Zebrafish Genes Related to Distal- Less: Part of a Homeobox Gene Code for the Head. *The Journal of Neuroscience, 14*(6), 3475-3486. doi:10.1523/JNEUROSCI.14-06-03475.1994

Krauss, S., Johansen, T., Korzh, V., & Fjose, A. (1991). Expression Pattern of Zebrafish Pax Genes Suggests a Role in Early Brain Regionalization. *Nature, 353*(6341), 267-270. doi:10.1038/353267a0

Manoli, M., & Driever, W. (2014). Nkx2.1 and Nkx2.4 Genes Function Partially Redundant During Development of the Zebrafish Hypothalamus, Preoptic Region, and Pallidum. *Frontiers in Neuroanatomy, 8*(DEC), 1-16. doi:10.3389/fnana.2014.00145

Martin, S. C., Heinrich, G., & Sandell, J. H. (1998). Sequence and Expression of Glutamic Acid Decarboxylase Isoforms in the Developing Zebrafish. *266*(October 1997), 253-266.

Nornes, S., Clarkson, M., Mikkola, I., Pedersen, M., Bardsley, A., Martinez, J. P., Krauss, S., & Johansen, T. (1998). Zebrafish Contains Two Pax6 Genes Involved in Eye Development. *Mechanisms of Development*.

Ryu, S., Mahler, J., Acampora, D., Holzschuh, J., Erhardt, S., Omodei, D., Simeone, A., & Driever, W. (2007). Orthopedia homeodomain protein is essential for diencephalic dopaminergic neuron development. *Curr Biol, 17*(10), 873-880. doi:10.1016/j.cub.2007.04.003

Tokumoto, M., Gong, Z., Tsubokawa, T., Hew, C. L., Uyemura, K., Hotta, Y., & Okamoto, H. (1995). Molecular Heterogeneity among Primary Motoneurons and within Myotomes Revealed by the Differential mRNA Expression of Novel Islet-1 Homologs in Embryonic Zebrafish. *Developmental Biology, 171*(2), 578-589. doi:10.1006/dbio.1995.1306

Toyama, R., Curtiss, P. E., Otani, H., Kimura, M., Dawid, I. B., & Taira, M. (1995). The LIM class homeobox gene lim5: implied role in CNS patterning in Xenopus and zebrafish. *Developmental Biology, 170*(2), 583-593. doi:10.1006/dbio.1995.1238
